# Temporomandibular joint disc repositioning and occlusal splint for adolescents with skeletal class II malocclusion: a single-center, randomized, open-label trial

**DOI:** 10.1186/s12903-023-03402-3

**Published:** 2023-09-27

**Authors:** Jiali Sun, Huimin Zhu, Chuan Lu, Jieyun Zhao, Xin Nie, Zhi Yang, Dongmei He

**Affiliations:** 1 Department of Oral Surgery, Ninth People’s Hospital, College of Stomatology, Shanghai Key Laboratory of Stomatology & Shanghai Research Institute of Stomatology, Shanghai Jiao Tong University School of Medicine, 639 Zhizaoju Road, Shanghai, 200011 China; 2 Biostatistics Office of Clinical Research Unit, Shanghai Ninth People’s Hospital, Shanghai Jiao Tong University School of Medicine, Shanghai, 200011 China; 3 Department of Oral and Cranio-maxillofacial Surgery, Shanghai Ninth People’s Hospital, College of Stomatology, Shanghai Key Laboratory of Stomatology & Shanghai Research Institute of Stomatology, Shanghai Jiao Tong University School of Medicine, 639 Zhi Zao Ju Road, Shanghai, 200011 China

**Keywords:** Temporomandibular joint, Anterior disc displacement, Disc repositioning, Class II malocclusion, Adolescent, Occlusal splints

## Abstract

**Background:**

Temporomandibular joint (TMJ) disc repositioning through open suturing (OSu) is a new disc repositioning method. Its result for adolescents with condylar resorption and dentofacial deformities combined with and without postoperative occlusal splints (POS) has not been well studied.

**Objective:**

This study was to evaluate and compare the effects of OSu with and without POS in the treatment of TMJ anterior disc displacement without reduction (ADDwoR) in adolescent skeletal Class II malocclusion.

**Methods:**

A total of 60 adolescents with bilateral ADDwoR were enrolled in this study. They were randomly allocated into two groups: OSu with and without POS. Magnetic resonance imaging (MRI) and lateral cephalometric radiographs were used to measure changes in condylar height and the degree of skeletal Class II malocclusion from before operation and at 12 months postoperatively. Changes in these indicators were compared within and between the two groups.

**Results:**

After OSu, both groups exhibited significant improvements in condylar height and occlusion at the end of 12 months follow-up (*P* < 0.05). The group of OSu with POS had significantly more new bone formation (2.83 ± 0.75 mm vs. 1.42 ± 0.81 mm, *P* < 0.001) and improvement in dentofacial deformity than the group of OSu only (*P* < 0.05). The new bone height was significantly correlated with POS (*P* < 0.001), the changes of SNB (*P* = 0.018), overjet (*P* = 0.012), and Wits appraisal (*P* < 0.001).

**Conclusion:**

These findings indicated that OSu can effectively stimulate condylar regeneration and improve skeletal Class II malocclusion in adolescents with bilateral ADDwoR. The results are better when combined with POS.

**Trial registration:**

This trial was prospectively registered on the chictr.org.cn registry with ID: ChiCTR1900021821 on 11/03/2019

**Supplementary Information:**

The online version contains supplementary material available at 10.1186/s12903-023-03402-3.

## Introduction

Temporomandibular joint (TMJ) anterior disc displacement (ADD) is one of the most prevalent TMJ disorders (TMD) in the population [1]. According to whether the disc can return to the normal position during mandibular movement, disc displacement is classified as ADD with reduction (ADDwR) or ADD without reduction (ADDwoR). During growth, ADD without treatment may cause condylar resorption and lead to a decrease in condylar height (CH), creating or worsening a dentofacial deformity [2–6]. According to Schellhas et al. and our previous studies, without treatment, unilateral ADD would cause mandibular deviation, while bilateral ADD would lead to skeletal Class II malocclusion demonstrated as mandibular retrognathia [2–7]. Conservative treatment often fails to prevent progressive condylar resorption [8–10], while disc repositioning (DR) by surgery could stimulate condylar regeneration, which was especially benefit for the correction of Class II malocclusion [3, 5, 6, 8, 11–15]. So far, there are 2 methods for DR [16–18]: suturing by arthroscopy or open suturing (OSu), and anchorage by a mini-screw anchor (MsA). Our previous study showed that OSu had more new bone formation than MsA in the treatment of adolescent ADDwoR [6].

After DR we found that the condyle moved anteriorly and inferiorly with increased joint space, which caused open bite in the molars. While after an average of 3 months postoperation, the condyle returned to its normal position [10, 19, 20]. We then used postoperative occlusal splint (POS) to maintain the open bite, so that the increased joint space after DR was benefit for condylar regeneration [15, 21]. In our previous studies, by magnetic resonance imaging (MRI) and cephalometric measurement, patients with POS after DR by MsA had more condylar new bone formation and improved dentofacial deformities when combined with orthodontic treatment than the one without POS [4, 13, 15].

Hence, we hypothesized that in adolescent skeletal Class II malocclusion with bilateral ADDwoR, DR by OSu with POS would stimulate more condylar bone regeneration and improve mandibular retrusion than surgery only. A randomized controlled trial was designed to evaluate this hypothesis.

## Materials and methods

### Study design

**Fig. 1:**
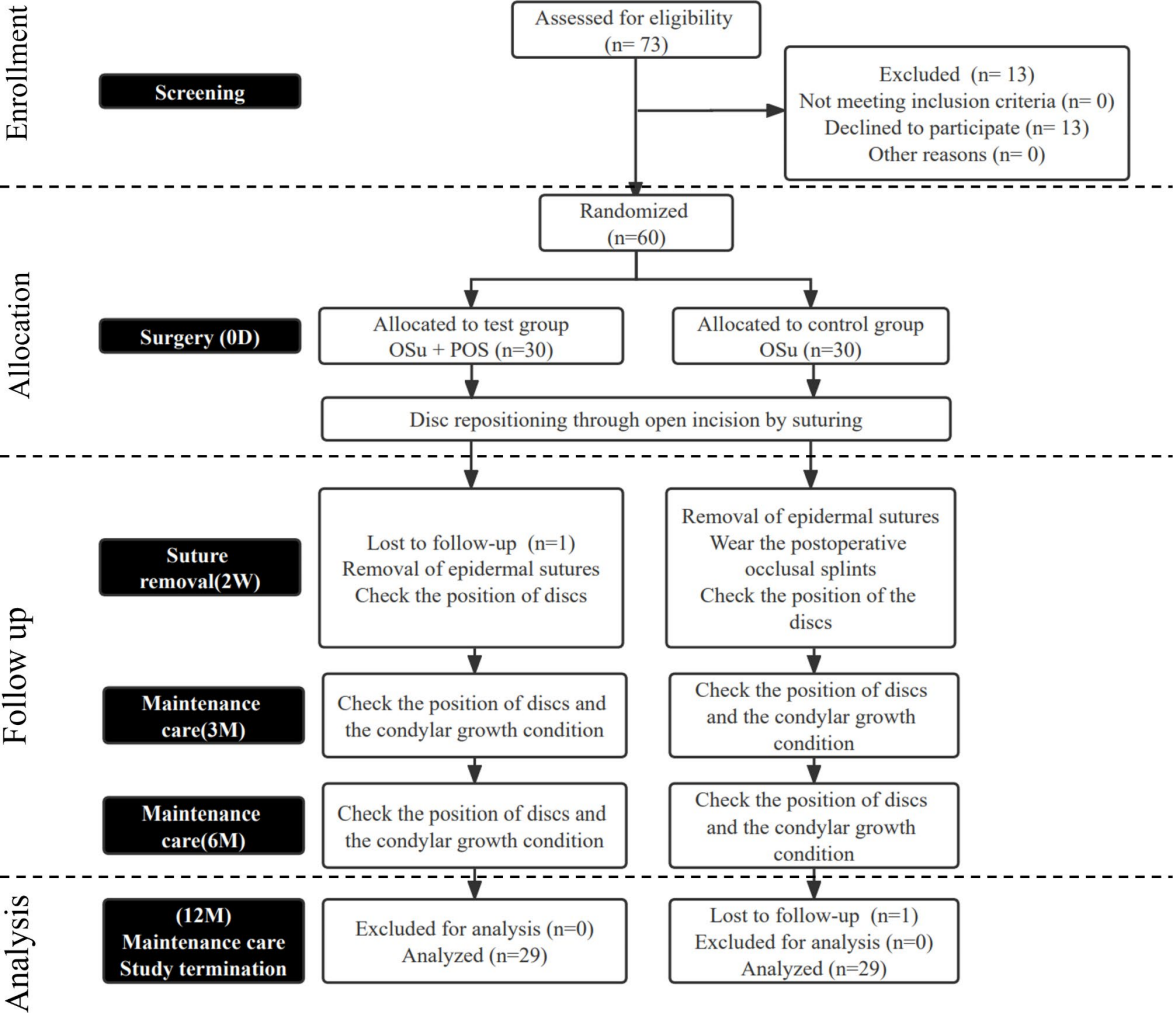
CONSORT flow diagram of participants in the trial

The trial was registered at the Chinese Clinical Trial Registry, a member of the World Health Organization international clinical trials registry (Registration Number: ChiCTR1900021821, Date: 11/03/2019). This study was approved by the Institutional Review Board of the Shanghai 9th People’s Hospital (JYLJ201805). All investigators participated in calibration meetings for standardizing the clinical trial protocol. It was conducted following ethical principles by the Declaration of Helsinki and good clinical practice guidelines. The flowchart for the CONSORT guidelines is presented in Fig. 1.

The trial was designed as a single-center, randomized, open-label, parallel-controlled clinical trial. Patients with bilateral ADDwoR and skeletal Class II malocclusion were recruited from the Department of Oral Surgery, Shanghai 9th People’s Hospital, from January 2019 to January 2021.

### Sample-size calculation

According to our previous retrospective study of ADD with skeletal Class II malocclusion treatment by MsA with and without POS [4], the minimum difference in new bone height of the condyle was calculated for the sample size of this study by OSu with and without POS. 24 patients in each group were estimated as a minimum sample size to detect a clinically relevant difference, with a power of 80% and a statistical significance level of 0.05 (two-tailed). Allowing for a dropout rate of 20%, the total sample size was determined to be 60 patients.

### Inclusion and exclusion criteria

The inclusion criteria were: (1) age between 12 and 20 years; (2) bilateral ADDwoR (Wilkes stage III to V) diagnosed by MRI; (3) skeletal Class II malocclusion with SNB < 78°and ANB > 4°measured by cephalometric films [22]. The exclusion criteria were: 1) history of TMJ surgical treatment and orthodontic treatment;2) history of developmental and/or systemic disorders such as autoimmune disease and rheumatoid arthritis that might affect craniofacial growth; 3) small condylar process or poor bone quality; (4) poorly formed articular disc length; (5) patients with malignant tumors and end-stage diseases; (6) pregnancy or lactation.

After the patients were screened, written informed consent to participate was obtained before enrolling them in the study.

### Randomization

The patients were given an enrollment number (by H.Z.) in matched sealed envelopes that included group assignments based on block randomization (fixed block size of 4) by an independent statistician (S.C.) from the Biostatistics Office. Each patient was allocated into one of the following groups immediately before the operation:

#### Test group (OSu + POS group):

DR through OSu and POS.

#### Control group (OSu group):

DR through OSu.

TMJ MRI images, lateral cephalometric films, and dentition were obtained before the operation. The TRIOS3 oral scanner (3shape, Denmark) was used to record the dentition for POS fabrication and further orthodontic treatment by the orthodontist (Z.Y).

### Surgical procedures

**Fig. 2:**
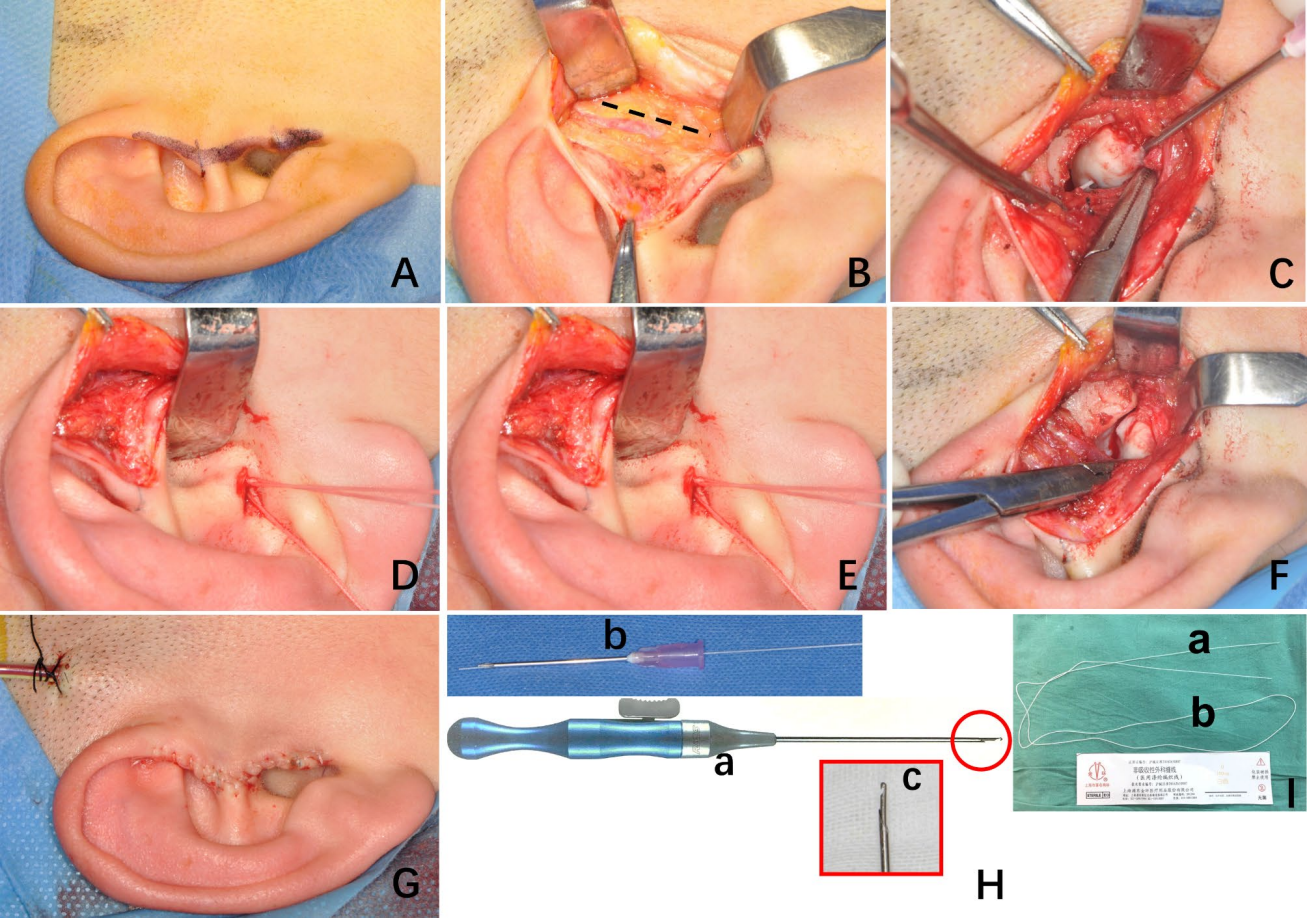
Steps of disc repositioning by OSu (**A**-**G**) and suture instruments (**H**-**I**). **A**-**B**, exposure of the operative area through a small modified pre-auricular approach; **C**-**G**, suturing method; **H**, (**a**) exchangeable hooked thread lifter and (**b**) 20 gauge needle, (**c**) Tip of the hook lifter; **I**, suture thread, (**a**) guiding zone, (**b**) functional zone

One senior surgeon (D.H.) performed DR by OSu under general anesthesia [18]. Through a small modified pre-auricular approach, the joint capsule was entered for disc repositioning. The disc was passively reduced after completely releasing the anterior attachment. Two horizontal sutures were placed at the border of the posterior band by a 20-gauged needle and a self-designed hook. The disc was sutured to the posterior articular capsule with 6 knots buried under the external auricular cartilage (Fig. 2).

### POS fabrication

**Fig. 3:**
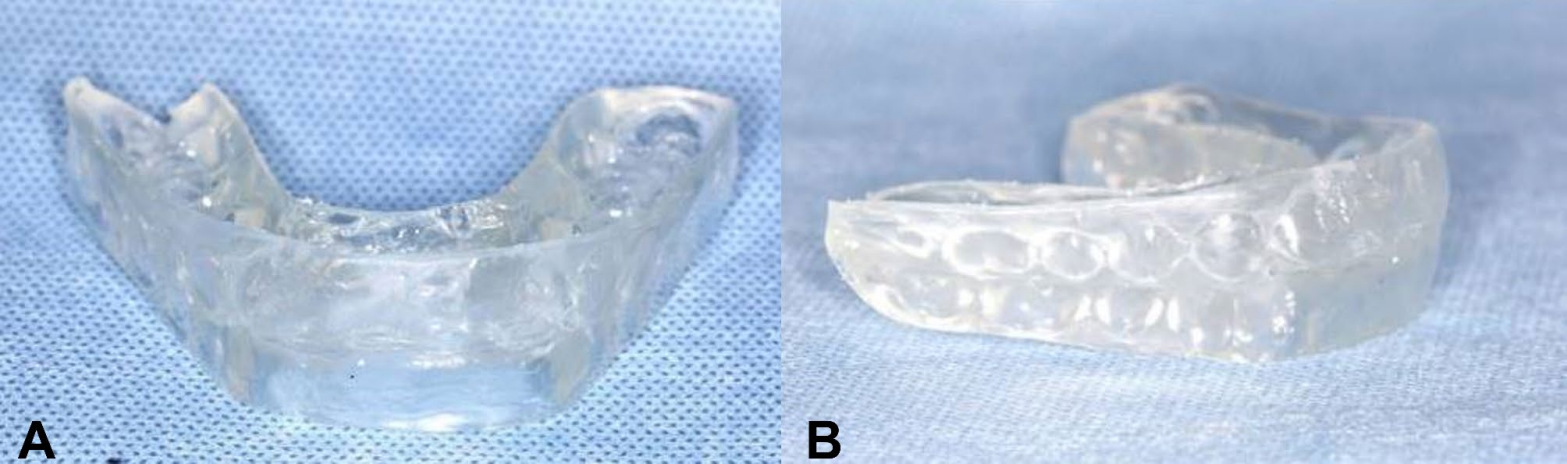
Postoperative occlusal splint. **A**, frontal view; **B**, lateral view

After operation, the condyle was moved forward and downward by the increased joint space, resulting in mandibular forward movement and posterior open bite [19]. An interocclusal wax registration was made to record this occlusion and sent to the orthodontist (Z.Y.) to design POS, so that new bone can be formed in the increased joint space and conducive to orthodontic treatment later. POS was fabricated by using polymethyl methacrylate material and provides complete coverage of the maxillary and mandibular dentition. Generally, the vertical dimension of the splint height is less than 2 mm. (Fig. 3). Patients were required to wear POS no less than 12 hours per day for 12 months [4].

MRI examination within one week after the operation was taken to assess the disc position. After 12 months, MRI and cephalometric films were taken for all patients to evaluate CH and jaw development. Patients were excluded if the discs were not properly repositioned or if they had relapsed. Adverse events of the DR and POS were monitored throughout the clinical trial period.

### Outcomes

**Fig. 4:**
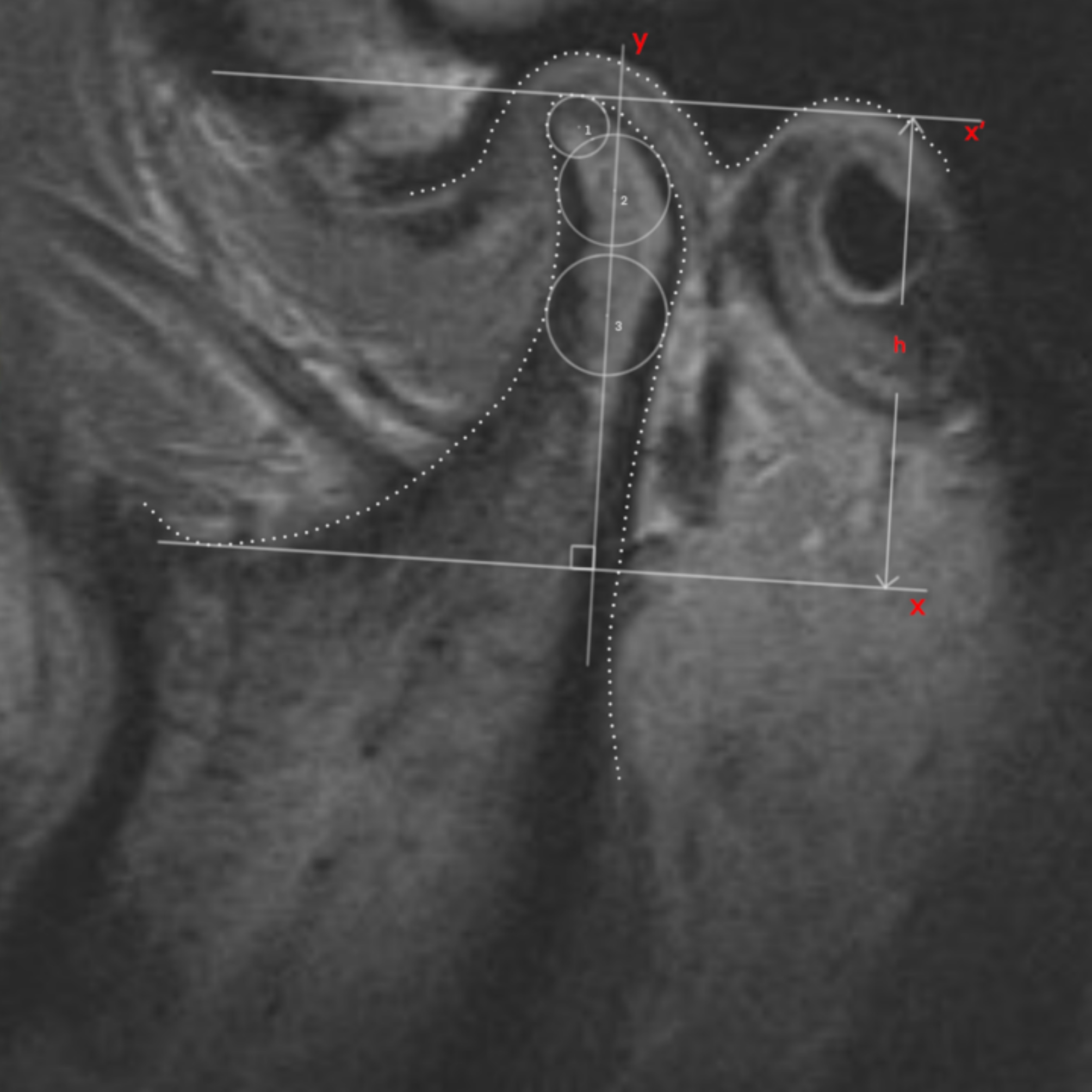
The condylar height measured by three-circle method in MRI

The primary outcome was condylar height measured by MRI (1.5-Tesla imager, Signa; General Electric, Milwaukee, WI, USA). Sagittal images with closed- and open-mouth positions and coronal images of the condyle were acquired for evaluation of disc position, condylar bone status and height [23, 24]. Images with the largest section of the condyle were selected, and the condylar height was determined based on the three-circle method [3]. E-Ruler measurement software was used to obtain parameter values with an accuracy of 0.01 mm (Fig. 4). Condylar height measurements before and at 12 months after operation were obtained. The positions of the generated new bone shown as double contour images were recorded as anterior, superior and posterior parts of the condyle.

**Fig. 5:**
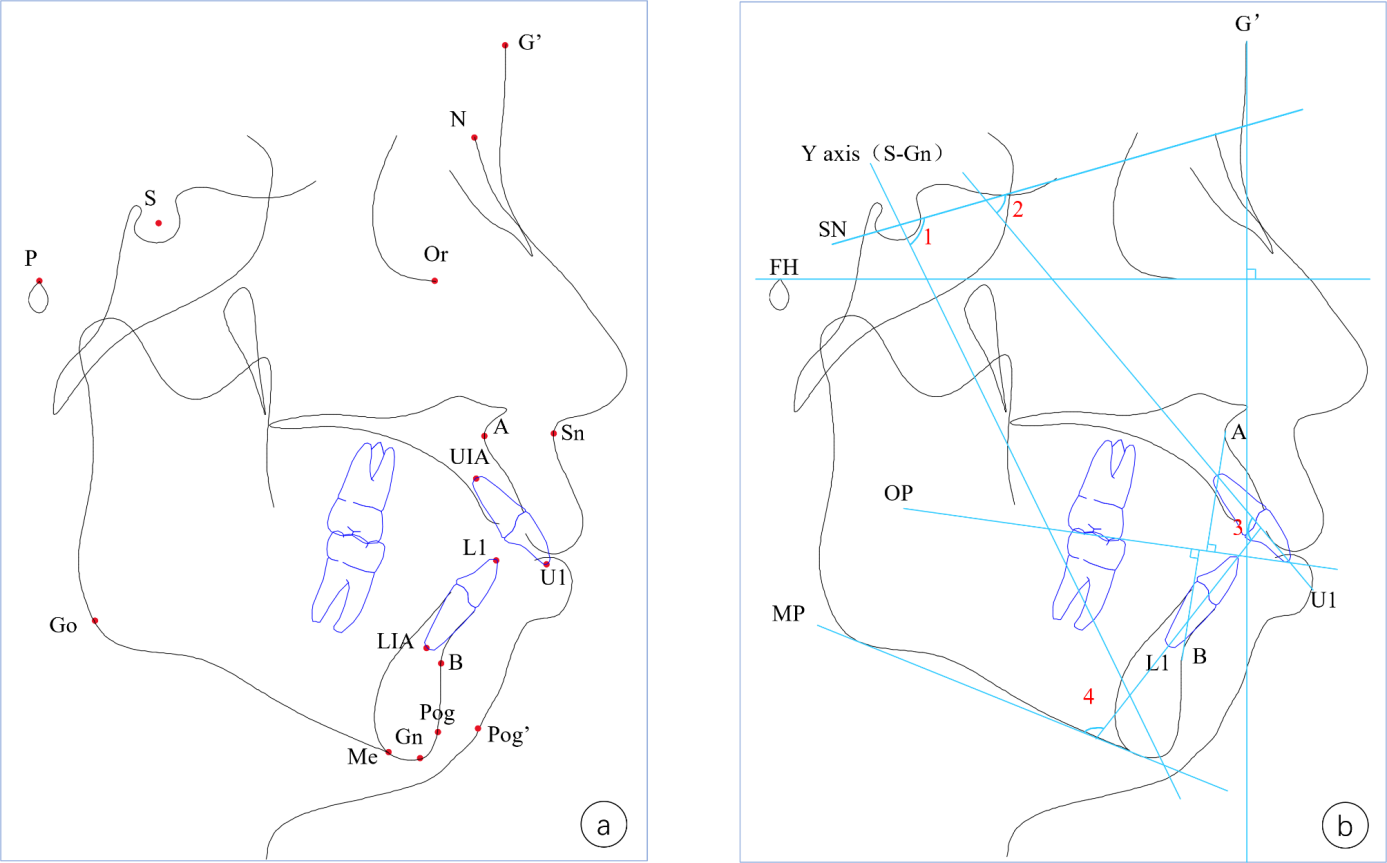
Cephalometric measurement. **a**, landmarks; **b**, planes and angles:1. Y-axis angle, 2. U1-SN, 3. U1-L1, 4. L1-MP.

The secondary outcome was the degree of skeletal Class II malocclusion measured by lateral cephalometric films, including the following variables: ∠SNA, ∠SNB, ∠ANB, incisor overjet, Wits appraisal, Y-Axis angle, Pog’-G’ (Pog’, pogonion of soft tissue; G’, a plane perpendicular to Frankfort horizontal plane through glabella), Sn-G’ (Sn, subnasale), U1-SN and, U1-L1. Shanghai Ninth People’s Hospital standard cephalometric analysis was used under Dolphin 11.5 software (Dolphin Imaging, Chatsworth, CA, USA) for measurement before operation and at 12-month follow-up (Fig. 5).

To guarantee the accuracy, MRIs and cephalometric radiographs were blindly evaluated by 2 clinicians (J.S & H.Z) twice at a 2-week interval. The average value of the 2 clinicians was calculated for comparison. If a statistical difference by a paired *t*-test was discovered (*P* < 0.05) in the 2 data sets, experts in TMJ or orthodontists were required to repeat the measurements.

### Statistical analysis

Two TMJs of each patient were included in the analysis. The analyses applied the intention-to-treat (ITT) principle by analyzing all available data from all randomized patients according to their treatment allocation. Continuous variables with normal distribution were presented as mean ± standard deviation (SD); non-normal variables were reported as median (interquartile range). Differences in changes in condylar heights, maxillofacial sagittal mandibular position, and occlusal relationship at pre-operation and 12 months post-operative were analyzed by paired *t*-tests. Student *t*-test (for continuous variables) or Chi-Squared test (for qualitative variables) were used for comparing the differences in the above indicators between the two groups. Pearson test (when the variables were normally distributed) and Spearman correlation test (when the variables were not normally distributed) were used to evaluate the correlation between the height of condylar new bone with POS and changes of the skeletal Class II malocclusion. P < 0.05 was considered statistically significant. IBM SPSS version 25.0 (IBM Corp., Armonk, NY, USA) was utilized to analyze all data.

## Results

One patient in the control group and one in the test group were lost because of Covid-19 outbreak, they refused to come for follow-up, so the trial procedures were completed with 58 patients (n = 29 in the test and control groups, respectively). At baseline, the mean age of the patients was 16.48 ± 2.45 years, 50 were females and 10 were males, and there were no statistical significances between the two groups in these or other baseline characteristics (*P* < 0.05, Table 1). None of the patients showed any side effects beyond the normal range for surgical procedures.

**Table 1:** Baseline demographic and radiographic characteristics of participants

	Control (n = 30)	Test (n = 30)
Average age (years)	16.30 (2.26)	16.67 (2.60)
Sex		
Men	6 (20.0%)	4 (13.3%)
Women	24 (80.0%)	26 (86.7%)
Wilkes stage		
III	10 (16.7%)	9 (15.0%)
IV	50 (83.3%)	51 (85.0%)

After DR, MRI showed that all discs were repositioned. In the OSu group, the condylar height increased from 19.11 ± 3.83 mm preoperatively to 20.52 ± 3.80 mm at the 12-month follow-up, with a mean increase of 1.42 ± 0.81 mm (*P* < 0.001). Among them, 46.6% of joints (27/58) had cap-shaped new bone formation in the anterior, superior, and posterior parts of the condyle. 89.7% joints (52/58) had new bone formation in any position. ∠SNB increased 0.65 ± 0.64° (*P* < 0.001), and other parameters decreased including incisal overjet (1.16 ± 1.03 mm, *P* < 0.001), ∠ANB (0.54 ± 1.18°, *P* = 0.020), Wits appraisal (0.55 ± 1.68 mm, *P* = 0.091), Y-axis angle (0.09 ± 1.26 °, *P* = 0.694), and Pog’-G’ (0.74 ± 1.86 mm, *P* = 0.040, Table 2).

**Table 2:** Measurement of the condylar height and jaw position before and 12 months after operation within the two groups

Group	Contents	Pre-Operation	Post-Operation	P value
OSu	Condylar height	19.11 ± 3.83	20.52 ± 3.80	< 0.001*
	Overjet (mm)	5.19 ± 1.81	4.03 ± 1.54	< 0.001*
	SNA (°)	80.96 ± 3.68	81.03 ± 3.68	0.643
	SNB (°)	76.08 ± 3.48	76.73 ± 3.55	< 0.001*
	ANB(°)	4.88 ± 1.87	4.33 ± 1.88	0.020*
	Wits appraisal (mm)	1.17 ± 2.87	0.62 ± 2.59	0.091
	Y-axis angle (°)	72.03 ± 5.24	71.93 ± 5.22	0.694
	U1-SN (°)	105.13 ± 8.23	105.56 ± 8.36	0.455
	Pog’-G’(mm)	8.75 ± 8.59	8.01 ± 7.72	0.040*
	Sn-G’ (mm)	4.65 ± 3.73	4.62 ± 3.30	0.916
	L1-MP	96.90 ± 6.23	97.98 ± 6.68	0.123
	U1-L1 (°)	118.21 ± 11.80	117.19 ± 10.92	0.232
OSu + POS	Condylar height	19.00 ± 3.65	21.83 ± 3.85	< 0.001*
	Overjet (mm)	5.30 ± 2.30	3.26 ± 1.85	< 0.001*
	SNA (°)	81.06 ± 3.06	81.44 ± 3.35	0.162
	SNB (°)	75.13 ± 3.92	76.96 ± 4.38	< 0.001*
	ANB(°)	5.81 ± 2.37	4.41 ± 2.57	< 0.001*
	Wits appraisal (mm)	1.79 ± 2.99	-0.65 ± 2.90	< 0.001*
	Y-axis angle (°)	74.85 ± 4.00	73.30 ± 4.57	0.001*
	U1-SN (°)	106.46 ± 7.79	106.36 ± 8.65	0.901
	Pog’-G’(mm)	11.74 ± 6.97	9.39 ± 6.12	0.001*
	Sn-G’ (mm)	3.70 ± 3.10	3.98 ± 3.07	0.073
	L1-MP	98.62 ± 7.66	100.03 ± 7.17	0.072
	U1-L1 (°)	113.02 ± 10.97	113.38 ± 10.46	0.672

Abbreviated: OSu, open suturing; POS, postoperative splint. *P < 0.05

**Fig. 6:**
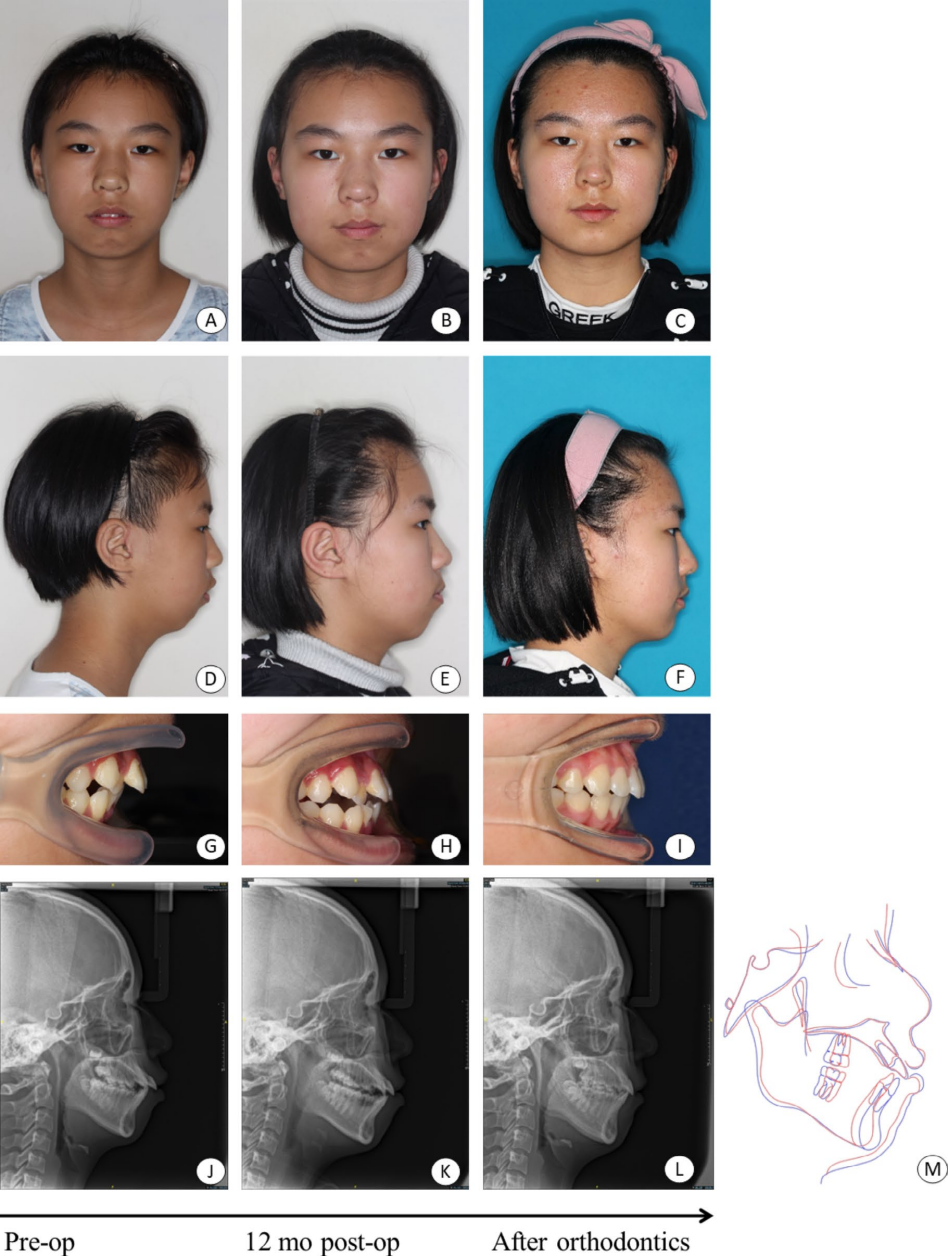
Female, 12years old, in the OSu + POS group. **A**, **D**, Preoperative facial photo; **B**, **E**, 12 months postoperative photo; **C**, **F**, photo after orthodontics treatment; **G**, preoperative overjet; **H**, 12 months postoperative overjet; **I**, overjet after orthodontic treatment; **J**, preoperative cephalometric film; **K**, 12 months postoperative cephalometric film; **L**, cephalometric film after orthodontic treatment; **M**, pre- (red outline) and 12 months postoperative (blue outline) cephalometric measurement showed improved mandibular retrusion

**Fig. 7:**
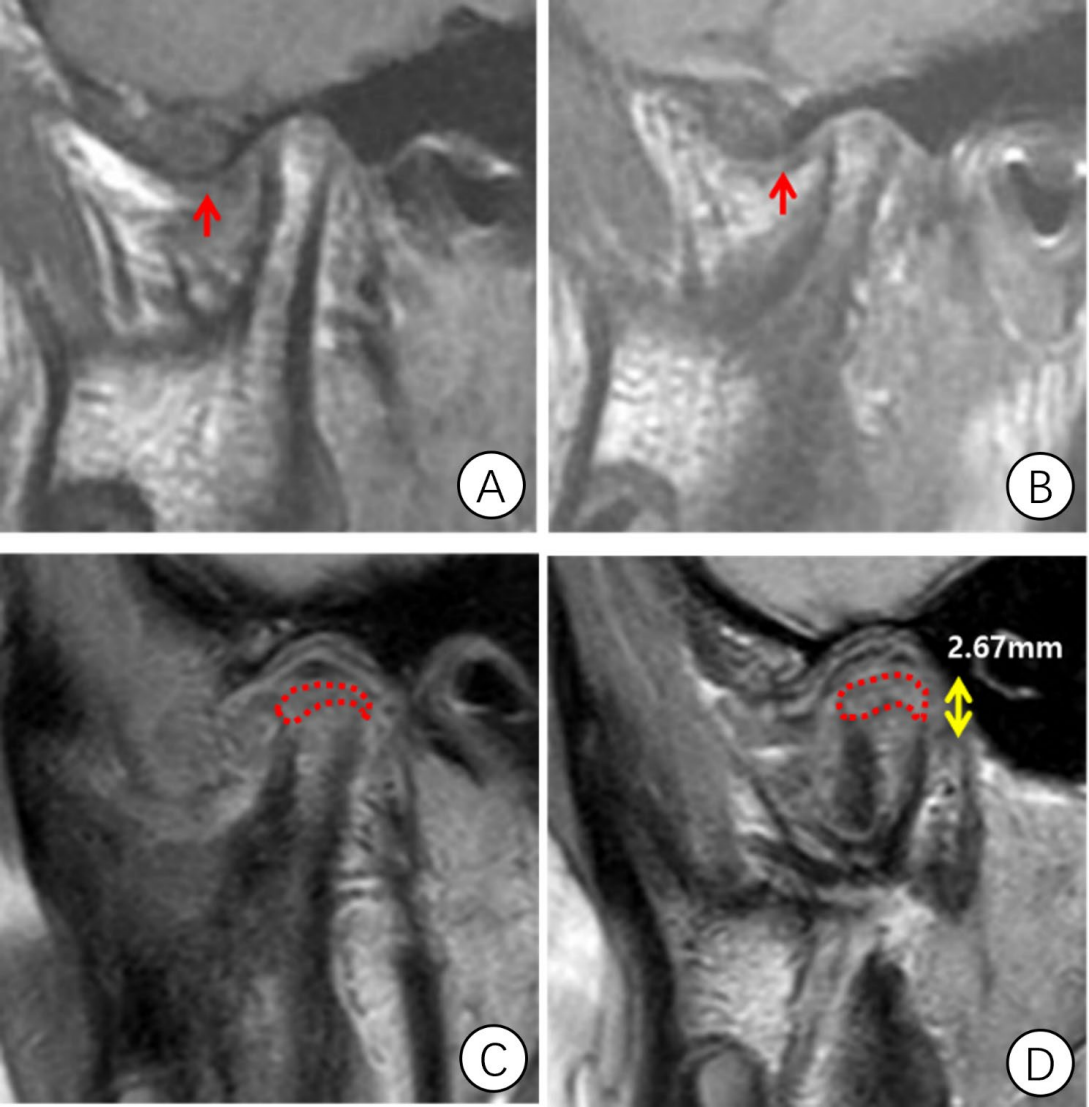
MRI of patient in Fig. 5. **A**, **B**, anteriorly displaced disc (red arrows), and discontinuous bone cortex of right & left condyles before operation; **C**, **D**, cap-shaped regenerated bone (red dashed line) after 12 months post operation

In the OSu with POS group, the condylar height increased from 19.00 ± 3.65 mm preoperatively to 21.83 ± 3.85 mm at a 12-month follow-up, with a mean increase of 2.83 ± 0.75 mm (*P* < 0.001). Among them, 74.1% of joints (43/58) had cap-shaped osteogenesis. 100% joints (58/58) had new bone formation in any position. ∠SNB increased 1.83 ± 1.19° (*P* < 0.001), and other parameters decreased including the incisal overjet (2.04 ± 1.71 mm, *P* < 0.001),∠ANB (1.40 ± 1.30°, *P* < 0.001), Wits appraisal (2.43 ± 1.94, *P* < 0.001), Y-axis angle (1.55 ± 2.30°, *P* = 0.001), and Pog’-G’ (2.36 ± 3.59 mm, *P* = 0.001, Table 2; Figs. 6 and 7).

There was no statistical difference in SNA, U1-SN, Sn-G’, L1-MP and U1-L1 between the two groups before and after treatment (*P* > 0.05, Table 3). OSu with POS group had significantly more regenerated condylar new bone height (*P* < 0.001), higher osteogenesis rates (100.0% vs 89.7%, χ^2^ test, *P* = 0.036) and cap-shaped new bone formation rates(74.1% vs 44.8%, χ^2^ test, *P* = 0.001), more increased SNB (*P* < 0.001), more decreased overjet (*P* = 0.020), ANB (*P* = 0.011), Wits appraisal (*P* < 0.001), Y-axis angle (*P* = 0.004), and Pog’-G’ (*P* = 0.038) than OSu only group (Tables 3 and 4).

**Table 3:** Difference of the condylar height and jaw position before and 12 months after operation between the two groups

Contents	Difference		P value
	OSu	OSu + POS	
Condylar height	1.42 ± 0.81	2.83 ± 0.75	< 0.001*
Overjet (mm)	-1.16 ± 1.03	-2.04 ± 1.71	0.020*
SNA (°)	0.07 ± 0.83	0.38 ± 1.44	0.318
SNB (°)	0.65 ± 0.64	1.83 ± 1.19	< 0.001*
ANB(°)	-0.54 ± 1.18	-1.40 ± 1.30	0.011*
Wits appraisal (mm)	-0.55 ± 1.68	-2.43 ± 1.94	< 0.001*
Y-axis angle (°)	-0.09 ± 1.26	-1.55 ± 2.30	0.004*
U1-SN (°)	0.43 ± 2.97	-0.10 ± 4.28	0.588
Pog’-G’(mm)	-0.74 ± 1.86	-2.36 ± 3.59	0.038*
Sn-G’ (mm)	-0.03 ± 1.58	0.28 ± 0.82	0.053
L1-MP	1.08 ± 3.67	1.41 ± 4.06	0.748
U1-L1 (°)	-1.02 ± 4.50	0.37 ± 4.61	0.251

Abbreviated: OSu, open suturing; POS, postoperative splint. *P < 0.05

**Table 4:** New bone site after disc repositioning by OSu ± post-operative splint treatment

Group	Condyles	New bone site	No.	%
OSu + POS	58	None	0	0
		Superior	3	5.2
		Posterior	0	0
		Anterior	0	0
		Anterior + Superior	4	6.9
		Anterior + Posterior	0	0
		Superior + Posterior	8	13.8
		Anterior + Superior + Posterior	43	74.1
OSu	58	None	6	10.3
		Superior	4	6.9
		Posterior	2	3.4
		Anterior	0	0
		Anterior + Superior	11	19.0
		Anterior + Posterior	1	1.7
		Superior + Posterior	8	13.8
		Anterior + Superior + Posterior	26	44.8

Abbreviated: OSu, open suturing; POS, postoperative splint

The correlation analysis showed that the height of condylar new bone was significantly correlated with wearing POS (r = 0.542, *P* < 0.001) and changes of mandibular position (△SNB, r = 0.311, *P* = 0.018; △overjet, r=-0.393, *P* = 0.002; △Wits appraisal, r=-0.498, *P* < 0.001, Table 5).

**Table 5:** Correlation between the height of the new condylar bone and the use of post-operative splints, changes in the position of the jaws

		±POS	△SNB	△overjet	△wits appraisal
△CH	r	0.542	0.311	-0.393	-0.498
	P	< 0.001*	0.018*	0.012*	< 0.001*

Abbreviated: CH, condylar height; POS, postoperative splint. *P < 0.05

## Discussion

The prevalence of ADD was observed to be three times higher during adolescence compared to other stages of life [25]. Roh et al. used MRI and reported that the incidence of condylar resorption was four times higher in patients with ADDwoR than in the general population [26]. When ADD happened in adolescents, condylar growth may be affected because of exceeding physiological loading. Many research have shown that adolescent ADDwoR and dentofacial deformities were closely connected [27–29]. Our previous studies by MRI follow-up also showed that condylar height decreased without treatment in adolescent patients with ADDwoR [2–4, 6, 13]. Mandibular deviation or retrusion was aggravated when ADD occurred unilaterally [3, 5] or bilaterally [4, 6, 11]. Functional appliances are usually used to correct patients with retrognathic mandibles [30–32]. However, it is noneffective for Class II malocclusion adolescents with bilateral ADDwoR and may increase the risk of condyle resorption thus exacerbate dentofacial deformities [4, 10]. Whether articular disc reduction by surgery can control or prevent condylar bone resorption, promote bone regeneration, and eventually obtain a stable joint has become the focus of many scholars.

Wolford et al. and our previous studies showed that DR could alleviate condylar bone resorption and encourage bone regeneration, especially in adolescents [8, 29]. Currently, there are two methods for DR: suturing by arthroscopy or open incision and anchorage by MsA. From the 1990s, many scholars attempted to use arthroscopic techniques to treat TMJ ADD, but the success rate was not high [26, 33]. In 2001, Wolford proposed implanting Mitek anchors into the condyle to fix the displaced articular disc [34]. Yang modified the technique with a self-designed MsA for easy reimplantation and retied the sutures [16]. The MRI follow-up studies showed that 10 months and 2 years stability was 98.6% and 95.3%, respectively [23, 35]. In a 5-year follow-up study, 89% of the discs were stable by MRI evaluation [36]. For patients with small condyles such as idiopathic condylar resorption and osteoporosis, it is difficult to implant the MsA. The dissection around the condyle may also affect the blood supply and increase the risk of condylar resorption [15]. In 2012, Yang reported an arthroscopic suturing method with self-designed instruments [17] that provided stable disc repositioning of 95.3% in 749 discs after 24-month follow up [37]. Although arthroscopic suturing method modified by Yang [17] without anchor implantation is suitable for hypoplastic condyles, the heavy equipment including arthroscope, light source, monitor and coblation device and difficult technique limit its wide application. Therefore, He et al. [18] proposed OSu technique by using the same self-designed hook of Yang’s arthroscopic surgery. It is easier to perform especially for the one with hyperplastic posterior bands or perforation [18]. Compared with MsA, OSu does not involve the insertion of anchor nails, the growth of the condyle may have the least mutual influence on surgical performance. Our previous study of 84 adolescents with ADD and Class II skeletal malocclusion, showed that OSu produced more condylar new bone formation and incisor overjet reduction than MsA [6]. A 1-year follow up study in 104 ADD adolescents from 10 to 18 years also showed that, through MRI image and cephalometric films, DR by OSu could promote condylar regeneration and alleviate maxillofacial deformity [11]. In this study, we determined the age of the participants according to the adolescence definition of World Health Organization (the second decade of life, 10–19 years of age). Although our sample population ranged wide from 12 to 20 years and most of them were women who ended growth after 15 years old [5, 6, 20, 38], at 1 year follow-up after DR, the condylar height increased significantly with improved dentofacial deformity. So it is the DR surgery that simulated condylar regeneration especially in adolescents who had strong tissue repair ability. By post-operative orthodontic treatment, orthognathic surgery may be avoided.

Another factor that affects postoperative condylar new bone formation is the application of POS. After DR, the condyle moved forward and downward, with the increasing of the superior and posterior joint space, resulting in posterior open bites. Without any interventions, the condyle returned to its normal position an average of 3 months after surgery [19]. Using POS can maintain this enlarged joint space, thus promoting new bone formation. It had been suggested that exceeding physiological loading could disrupt joint lubrication, leading to temporary hypoxia and consequently fibrocartilage destruction [39, 40]. So, wearing POS helps protect the new fragile bone from absorption under stressful conditions. Our previous studies showed that after DR by either arthroscopy or open surgery with POS, CH and dentofacial deformity improved more than without POS [4–6, 15]. We found that new bone remodeling generally occurs around 3 months after DR, with a peak around 6 months. Compared with OSu only, POS group had significantly more condylar new bone formation (2.83 ± 0.75 mm vs. 1.42 ± 0.81 mm) and a higher rate of cap-shaped osteogenesis (74.1% vs. 44.8%). Furthermore, the amount of condylar new bone was significantly correlated with POS and changes of the skeletal Class II malocclusion. In this study, orthodontic treatment began 1 year after DR for further malocclusion correction. The increased CH benefited orthodontic treatment especially in OSu with POS group.

It is debatable whether a patient will benefit from early open joint operations or whether a diverse variety of conservative nonsurgical methods should be considered before deciding on open joint surgery [41]. According to Gonçalves et al., a period of symptoms within 4 years provides the best predictor of outcomes in TMJ disc surgery [42]. Nitzan and Marmary [43] discovered that the endurance of symptoms had a detrimental impact on articular function. Our previous research has shown that ADD without intervention causes condylar resorption and may cause or accelerate dentofacial deformity in adolescent patients with and without TMJ symptoms. Under a comprehensive consideration, ADD should be treated as early as possible rather than as an end-stage disease later. Early surgery, particularly in adolescents during the growth spurt, should be conducted as soon as feasible to restore the condylar development potential, hopefully reducing the chance of future orthognathic surgery for mandibular retraction. Larger sample size is needed to determine whether DR by OSu and POS with further orthodontic treatment can avoid future orthognathic surgery.

## Conclusion

This randomized controlled trial demonstrated that DR by OSu with POS can stimulate more condylar new bone formation in adolescents than OSu only, which can better improve skeletal Class II malocclusion.

### Electronic supplementary material

Below is the link to the electronic supplementary material.

Supplementary Material 1

## Data Availability

All data generated or analysed during this study are included in this published article and its supplementary information files (Additional file 1).

## List of abbreviations

ADDwoR: Anterior disc displacement without reduction
ADDwR: Anterior disc displacement with reduction
CH: Condylar height
DR: Disc repositioning
ITT: Intention-to-treat
MRI: Magnetic resonance imaging
MsA: Mini-screw anchor
OSu: Open suturing
POS: Postoperative splints
SD: Standard deviation
TMD: Temporomandibular joint disorders
TMJ: Temporomandibular joint
